# Characteristics of pulmonary artery strain assessed by cardiovascular magnetic resonance imaging and associations with metabolomic pathways in human ageing

**DOI:** 10.3389/fcvm.2024.1346443

**Published:** 2024-02-29

**Authors:** Hongzhou Zhang, Shuang Leng, Fei Gao, Jean-Paul Kovalik, Hai Ning Wee, Kee Voon Chua, Jianhong Ching, John C. Allen, Xiaodan Zhao, Ru-San Tan, Qinghua Wu, Tim Leiner, Angela S. Koh, Liang Zhong

**Affiliations:** ^1^ Department of Cardiovascular Medicine, First Affiliated Hospital of Gannan Medical University, Ganzhou, Jiangxi, China; ^2^ National Heart Research Institute Singapore, National Heart Centre Singapore, Singapore, Singapore; ^3^ Duke-NUS Medical School, Singapore, Singapore; ^4^ Department of Endocrinology, Singapore General Hospital, Singapore, Singapore; ^5^ KK Research Centre, KK Women’s and Children’s Hospital, Singapore, Singapore; ^6^ Department of Cardiology, The Second Affiliated Hospital of Nanchang University, Nanchang, Jiangxi, China; ^7^ Department of Radiology, Mayo Clinic, Rochester, MN, United States

**Keywords:** pulmonary artery, metabolomics, ageing, cardiovascular magnetic resonance, strain

## Abstract

**Background:**

Pulmonary artery (PA) strain is associated with structural and functional alterations of the vessel and is an independent predictor of cardiovascular events. The relationship of PA strain to metabolomics in participants without cardiovascular disease is unknown.

**Methods:**

In the current study, community-based older adults, without known cardiovascular disease, underwent simultaneous cine cardiovascular magnetic resonance (CMR) imaging, clinical examination, and serum sampling. PA global longitudinal strain (GLS) analysis was performed by tracking the change in distance from the PA bifurcation to the pulmonary annular centroid, using standard cine CMR images. Circulating metabolites were measured by cross-sectional targeted metabolomics analysis.

**Results:**

Among *n* = 170 adults (mean age 71 ± 6.3 years old; 79 women), mean values of PA GLS were 16.2 ± 4.4%. PA GLS was significantly associated with age (*β *= −0.13, *P *= 0.017), heart rate (*β *=* *−0.08, *P *= 0.001), dyslipidemia (*β *=* *−2.37, *P *= 0.005), and cardiovascular risk factors (*β *=* *−2.49, *P *= 0.001). Alanine (*β *=* *−0.007, *P *= 0.01) and proline (*β *=* *−0.0009, *P *= 0.042) were significantly associated with PA GLS after adjustment for clinical risk factors. Medium and long-chain acylcarnitines were significantly associated with PA GLS (C12, *P *= 0.027; C12-OH/C10-DC, *P *= 0.018; C14:2, *P *= 0.036; C14:1, *P *= 0.006; C14, *P *= 0.006; C14-OH/C12-DC, *P *= 0.027; C16:3, *P *= 0.019; C16:2, *P *= 0.006; C16:1, *P *= 0.001; C16:2-OH, *P *= 0.016; C16:1-OH/C14:1-DC, *P *= 0.028; C18:1-OH/C16:1-DC, *P *= 0.032).

**Conclusion:**

By conventional CMR, PA GLS was associated with aging and vascular risk factors among a contemporary cohort of older adults. Metabolic pathways involved in PA stiffness may include gluconeogenesis, collagen synthesis, and fatty acid oxidation.

## 1 Introduction

Similar to the aorta, the pulmonary artery (PA) is susceptible to vascular remodeling against a background of age- and risk factor-related insults over time (1–3). In the PA, age-related vascular remodeling increases PA pressure due to decreased vascular compliance (2, 4, 5), leading to diseases such as pulmonary arterial hypertension (PAH) and heart failure.

While age-associated increases in PA pressure and diameter are well appreciated, PA stiffness in aging is rarely characterized. PA stiffness has been largely reported among clinical cardiovascular disease cohorts, utilizing advanced techniques such as invasive hemodynamics (6) or surrogate measures via echocardiogram (7–9). These methods are inherently disadvantageous for understanding PA stiffness in non-disease cohorts as they are invasive, variable—due to short transit time in the pulmonary trunk—or rely on complicated phase-contrast imaging, making them impractical for community-based studies. Among aging cohorts that may have unstable renal function, non-contrast-enhanced conventional cardiovascular magnetic resonance (CMR) appears advantageous over other techniques.

PA stiffness is a key disturbance associated with poor outcomes in both left and right heart diseases (10–13). Underscoring the important mechanical relationship between the PA and right ventricle (RV), the movement of the pulmonary valve plane arising from RV contraction causes longitudinal movement of the PA. Together with the expansibility of the artery, longitudinal movement contributes to PA deformation over a cardiac cycle. As the PA is mechanically connected to the RV, assessing PA stiffness across the longitudinal length of the PA may be useful for understanding diseases of the right heart (10, 13). This might be especially important in the absence of elevated pulmonary pressures, which calls for more sensitive tools to ascertain the state of PA stiffness before hemodynamic pressures rise.

Evidence-based therapies for the field of pulmonary hypertension remain less developed compared to therapeutics for left heart diseases, such as left ventricular heart failure. A key barrier lies in insufficient mechanistic understanding of the pathogenesis of pulmonary arterial diseases at the molecular level in human cohorts. Mapping molecular signals to sensitive quantitative measures of arterial properties can potentially facilitate exploratory interrogation of the complex vascular biology mechanisms that underpin the development of symptomatic vascular disease. The feasibility of this approach is supported by mechanistic studies that have shown changes in circulating metabolic intermediates in subjects with asymptomatic arterial stiffness (14, 15) as well as in subjects with PAH (16, 17). Whether disruptions in metabolic pathways, as indicated by metabolic intermediates, are observed in the pulmonary vasculature remains under-investigated.

With these considerations in mind, we proposed a method for assessing PA global longitudinal strain (GLS) based on CMR feature tracking methods and demonstrated significant correlations between PA GLS and surrogates of PA stiffness, including PA relative area change and pulse wave velocity (PWV) (18). Analogous to GLS of the ascending aorta as a measure of aortic stiffness (19), we further hypothesize that PA GLS obtained by this method may be associated with clinical factors such as age and vascular risk factors that alter PA stiffness. Guided by our prior work that detected associations between vascular stiffness and targeted metabolites in the acylcarnitines and related pathways, we studied serum metabolomics in conjunction with PA GLS to discover key metabolic pathways involved in PA stiffness.

## 2 Materials and methods

### 2.1 Study population

One hundred and seventy subjects were recruited from the Cardiac Aging Study, a prospective study initiated in 2014 that examines characteristics and determinants of cardiovascular function in older adults (20) without known cardiovascular disease. All participants had no self-reported history of physician-diagnosed cardiovascular diseases (such as coronary heart disease, atrial fibrillation), stroke, or cancer. The study adhered to the principles outlined in the Declaration of Helsinki, and the SingHealth Centralised Institutional Review Board approved the study protocol. Participants provided written informed consent upon enrollment.

All participants were examined and interviewed during a single study visit. They completed a standardized questionnaire that included medical history and coronary risk factors. Hypertension was defined by the current use of antihypertensive drugs or physician-diagnosed hypertension. Diabetes mellitus was defined by the current use of anti-diabetic agents or physician-diagnosed diabetes mellitus. Dyslipidemia was defined by the current use of lipid-lowering agents or physician-diagnosed dyslipidemia. Smoking history was categorized as ever smokers (former or current smoking) or never smokers. Sinus rhythm status was ascertained through a resting electrocardiogram. Clinical data were obtained on the same day as the assessment of CMR imaging and serum collection.

A validated non-exercise prediction model was employed to estimate peak oxygen uptake, VO_2_ (ml/kg/min) (21). The physical activity questionnaire included the frequency of exercise, length of time, and intensity for each workout. This model is closely linked to specific measures of cardiovascular structure and function (22).

### 2.2 CMR acquisition

**Figure 1:**
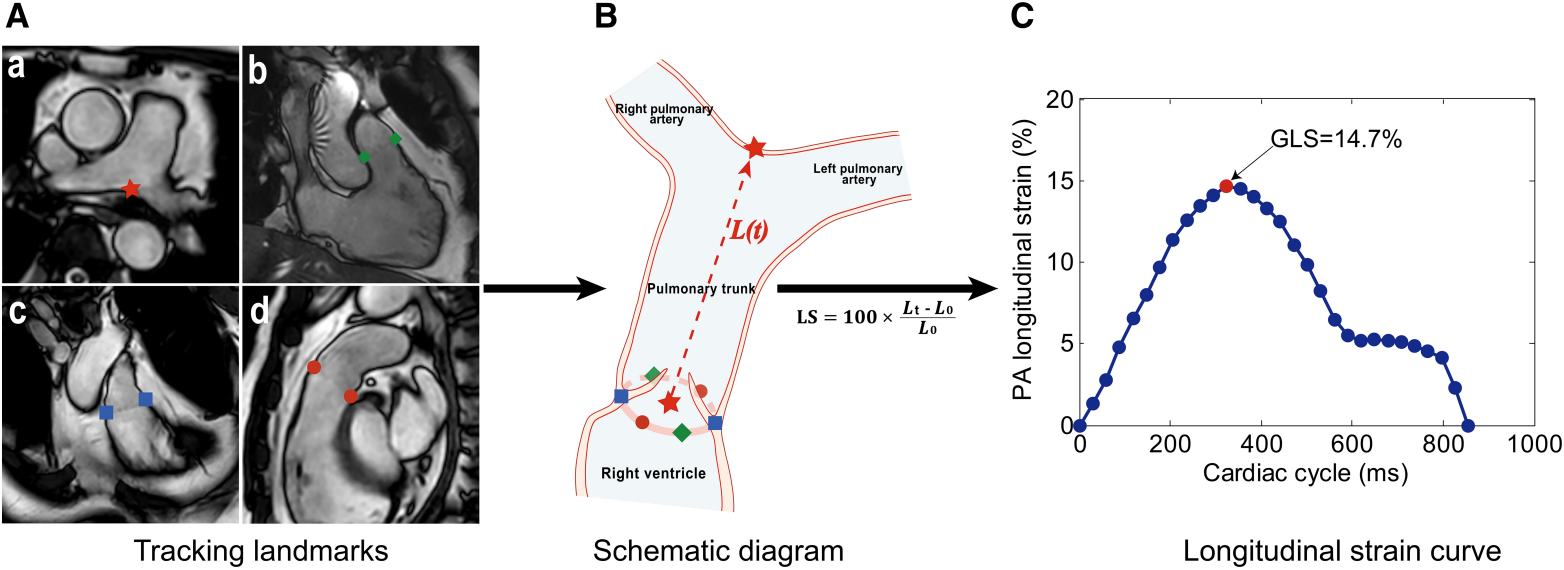
Measurement method of pulmonary artery (PA) global longitudinal strain (GLS). (**A**) Landmark tracking on the PA bifurcation (a, red star), right ventricular (RV) 3-chamber (b, green diamonds), coronal RV outflow tract (RVOT) (c, blue squares), and RVOT (d, red dots) views. (**B**) Schematic diagram of feature tracking. The trajectory of the centroid (red star) derived from six pulmonary annular points was calculated. The distance [*L(t)*, the red dotted line] between two red stars was automatically tracked throughout the cardiac cycle in 3D space. (**C**) Longitudinal strain (LS) curve. LS at a time point (*t*) relative to the initial time point (*t* = 0) at end-diastole was calculated as 100 × (*L_t_* –* L_0_*)/*L_0_*, and the PA GLS was defined as the maximal absolute strain value.

Cine CMR scans were performed using a balanced fast field echo sequence. All subjects were imaged on a 3 T magnetic resonance imaging system (Ingenia, Philips Healthcare, The Netherlands). The CMR acquisition protocol and typical parameters were published previously (23). Standard end-expiratory breath-hold cine images were obtained using a steady-state free precession pulse sequence, retrospective electrocardiographic gating, and a typical temporal resolution of 30 frames per cardiac cycle. These images were acquired in the PA bifurcation, RV outflow tract (RVOT), coronal RVOT, and RV 3-chamber views (Figure 1A). Additionally, end-expiratory breath-hold cine images were acquired in multi-planar long-axis views, including 2-, 3-, and 4-chamber views.

### 2.3 PA global longitudinal strain

PA GLS was assessed by one operator (H.Z.Z.) using an in-house semi-automatic algorithm (18) while remaining blinded to the clinical characteristics of participants and other CMR measurements. The motion of the PA bifurcation and the pulmonary valve annulus was automatically tracked over the cardiac cycle in PA bifurcation, RVOT, coronal RVOT, and RV 3-chamber views (Figure 1A). The trajectory of the centroid derived from six pulmonary annular points was obtained. The distance (*L*) from PA bifurcation to the centroid was calculated throughout the cardiac cycle. Longitudinal strain at a time point (*t*) relative to the initial time point (time 0) at end-diastole was calculated as 100 × (*L_t_*–*L_0_*)/*L_0_* (Figure 1B), and the PA GLS was defined as the maximal absolute strain value (Figure 1C). Details on the measurement of PA GLS can be found in the Supplementary Material.

Reproducibility was assessed in 20 randomly selected subjects. Inter-observer variability was evaluated by two independent operators (H.Z.Z. & S.L.), while intra-observer variability was assessed twice, with an interval of one month, by the first operator (H.Z.Z.). Correlation and Bland-Altman analyses were conducted to investigate intra- and inter-observer agreement.

### 2.4 RV function and global longitudinal strain

RV function was assessed by cardiologists blinded to other CMR measurements. The endocardium of the RV was automatically tracked on short-axis cine images at end-systole and end-diastole to obtain RV end-diastolic volume (EDV) and end-systolic volume (ESV), and to calculate stroke volume (SV) and ejection fraction (EF). Using cine 4-chamber CMR images, the RV endocardium was tracked by one reader (X.D.Z.), who was blinded to all participant characteristics. RV GLS was automatically obtained using dedicated, validated QStrain software (Version 2.0, Medis BV, Leiden, The Netherlands).

### 2.5 Metabolomic profiling

#### 2.5.1 Blood collection and serum processing

Antecubital venous blood samples (20–30 ml) were collected in the morning from consenting participants; fasting was not required. After collection, the blood samples were immediately placed on ice for transportation and processed within 6 h to obtain serum samples. The serum metabolomic profiling analysis was conducted at the Duke-NUS Metabolomics Facility.

#### 2.5.2 Targeted metabolomics profiling

Serum samples (50 μl) were spiked with a 10 μl deuterium labelled amino acid mixture and diluted with 400 μl methanol. After centrifugation of the mixture at 17,000 g for 5 min at 4°C, the supernatant fraction (10 μl) was collected for amino acid analysis. A pooled quality control sample was prepared by mixing equal amounts (10 μl) of each extracted serum sample. Extraction and measurement of amino acid panels (quantified in units of μM) were performed as previously described (24). Analysis was done on the MultiQuant™ 3.0.3 software (AB Sciex, DC, USA). For acylcarnitines, serum samples (100 μl) were similarly prepared as the amino acid analysis, but were instead spiked with 20 μl deuterium-labelled acyl-carnitine mixture and diluted with 800 µl methanol. Extraction and measurement of acyl-carnitine were performed as previously described (25). Data acquisition and analysis were conducted on an Agilent MassHunter Workstation B.06.00 Software.

During initial data analysis, standard analytical chemistry procedures were employed to assess data quality, including accuracy and precision. Accuracy was determined through low and high concentration quality control runs against the standard calibration curve. A sample of the pooled biologic quality control sample was measured repeatedly during the sample run to detect drift in the signal and assess precision. Additionally, the coefficient of variation was evaluated for each analyte in the pooled biologic quality control runs. Analytes with a coefficient of variation greater than 20% were excluded from further interpretation.

### 2.6 Statistical analysis

Clinical characteristics are presented as mean and standard deviation (SD) for continuous data and frequency and percentage for categorical data. Cardiovascular risk factor 2 (CVRF2) was defined as the presence of any two or more cardiovascular risk factors (hypertension, dyslipidemia, ever smoked).

The association between PA GLS and clinical risk factors was assessed using linear regression. All clinical risk factors that showed an association with PA GLS at *P *< 0.05 in univariate analysis were entered into a multiple linear regression analysis that related PA GLS to significant clinical risk factors, adjusting for metabolite effects.

The identification of metabolites associated with PA GLS occurred in two steps. First, simple linear regression with PA GLS was conducted to identify significant metabolites (*P *< 0.05). Second, multiple linear regression was performed on each metabolite associated with PA GLS (*P *< 0.05 in univariate analysis), adjusting for the effects of identified significant clinical risk factors.

Subjects were assigned to groups with higher or lower than average PA GLS—i.e., “high PA GLS” and “low PA GLS”, respectively—based on sex-specific mean values (16.3% for females and 16.1% for males). A heatmap, visualizing the normalized intensities of metabolites, was generated using Mass Profiler Professional software (Agilent, USA).

All statistical analyses were conducted using STATA 15 (College Station, Texas, USA). For all analyses, a two-tailed *P*-value of <0.05 was considered significant.

## 3 Results

We analyzed 170 participants (mean ± SD age 71 ± 6.3 years; 79 women) to obtain PA GLS (mean ± SD 16.2 ± 4.4%). The most common associated comorbidities of participants were hypertension (51.8%), diabetes mellitus (48.2%), dyslipidemia (20%), and smoking (18.2%). Additionally, 25.3% of participants had two or more risk factors (hypertension, dyslipidemia, ever smoked) (Table 1). All participants were in New York Heart Association Class I and were in sinus rhythm. Mean VO_2_ levels were 34 ± 5.9 ml/kg/min for the whole cohort, 37.7 ml/kg/min for men, and 29.9 ml/kg/min for women.

**Table 1:** Demographics and clinical characteristics of the study population.

Parameters	Overall (*n* = 170)
Age (year)	71 ± 6.3
Female, *n* (%)	79 (46.5)
Weight (kg)	60 ± 9.6
Height (cm)	159 ± 7.6
Body surface area (m^2^)	1.6 ± 0.2
Body mass index (kg/m^2^)	24 ± 3.2
SBP (mmHg)	147 ± 15.7
DBP (mmHg)	75 ± 10.9
Heart rate (beats/min)	74 ± 12.7
Hypertension, *n* (%)	88 (51.8)
Dyslipidemia, *n* (%)	34 (20)
Diabetes mellitus, *n* (%)	82 (48.2)
Smoking, *n* (%)	31 (18.2)
CVRF2, *n* (%)	43 (25.3)
VO_2_ (ml/kg/min)	34 ± 5.9
PA GLS (%)	16.2 ± 4.4
RV function	
RV EDV index (ml/m^2^)	68 ± 14.4
RV ESV index (ml/m^2^)	27 ± 8.9
RV SV index (ml/m^2^)	42 ± 7.7
RV EF (%)	62 ± 6.9
RV GLS (%)	−31 ± 5.5

Values are presented as mean ± SD or *n* (%).

SBP, systolic blood pressure; DBP, diastolic blood pressure; CVRF2, cardiovascular risk factor ≥2 (hypertension, dyslipidemia, ever smoked); VO_2_, oxygen uptake; PA, pulmonary artery; GLS, global longitudinal strain; RV, right ventricular; EDV, end-diastolic volume; ESV, end-systolic volume; SV, stroke volume; EF, ejection fraction.

PA GLS analysis was successfully performed in all subjects. The intra- and inter-observer correlation coefficients were *r *= 0.94 and *r *= 0.91, respectively, while the intra- and inter-observer coefficients of variation were 4.4% and 5.0%, respectively (Supplementary Figure S1).

Univariate linear regression was performed on PA GLS with clinical parameters as dependent variables (Table 2). Age, heart rate, dyslipidemia, smoking, and CVRF2 were negatively associated with PA GLS. Additionally, RV EDV index (*β *= 0.10, *P *= 0.001) and RV ESV index (*β *=* *0.12, *P *= 0.002) showed positive associations with PA GLS. However, there was no significant correlation between RVEF or RV GLS and PA GLS. PA GLS correlated with aerobic capacity among women (*r *= 0.31, *P *= 0.0049), but not among men (*r *= 0.038, *P *= 0.723).

**Table 2:** Summary of univariate linear regression coefficients for PA GLS regressed on demographic and clinical variables.

	*β* (95% CI)	*P-*value
Age	−0.13 (−0.23, −0.02)	**0**.**017**
Female	0.22 (−1.12,1.56)	0.75
Weight	0.04 (−0.03, 0.11)	0.28
Height	0.03 (−0.06, 0.12)	0.52
Body surface area	2.46 (−1.90, 6.82)	0.27
Body mass index	0.10 (−0.11, 0.31)	0.34
SBP	−0.03 (−0.07, 0.01)	0.17
DBP	−0.03 (−0.09, 0.03)	0.33
Heart rate	−0.08 (−0.14, −0.03)	**0**.**001**
Hypertension	0.02 (−1.31, 1.36)	0.98
Dyslipidemia	−2.37 (−4.0, −0.75)	**0**.**005**
Diabetes mellitus	−0.22 (−1.56, 1.11)	0.75
Smoking	−2.04 (−3.74, −0.34)	**0**.**019**
CVRF2	−2.49 (−3.98, −1.003)	**0**.**001**
VO_2_	0.07 (−0.04, 0.19)	0.20
RV EDV index	0.10 (0.06, 0.15)	**0**.**001**
RV ESV index	0.12 (0.06, 0.19)	**0**.**002**
RV SV index	0.20 (0.12, 0.28)	**<0**.**001**
RV EF	−0.05 (−0.14, 0.05)	0.35
RV GLS	0.07 (−0.06, 0.19)	0.30

SBP, systolic blood pressure; DBP, diastolic blood pressure; CI, confidence interval; CVRF2, cardiovascular risk factor ≥2 (hypertension, dyslipidemia, ever smoked); VO_2_, oxygen uptake; PA, pulmonary artery; GLS, global longitudinal strain; RV, right ventricular; EDV, end-diastolic volume; ESV, end-systolic volume; SV, stroke volume; EF, ejection fraction.

*P* values less than 0.05 are highlighted in bold.

We analyzed 86 metabolites, including 69 acylcarnitines and 17 amino acid metabolites. The list of measured metabolites is presented in Supplementary Table S1.

Linear regression analysis revealed associations between amino acids and PA GLS (Table 3). In univariate analysis, PA GLS was associated with alanine (*β *=* *−0.009, *P *= 0.001) and proline (*β *=* *−0.01, *P *= 0.008). Other amino acids showed no association with PA GLS. In multiple regression analysis between individual amino acids and PA GLS, adjusting for prior clinical covariates, both alanine and proline remained significantly associated with PA GLS.

**Table 3:** Summary of regression coefficients for PA GLS regressed on amino acids.

Amino acids	Unadjusted (Univariate linear regression)		Adjusted (Multiple linear regression)^a^	
	β (95% CI)	*P-*value	β (95% CI)	*P-*value
Alanine	−0.009 (−0.014, −0.004)	**0**.**001**	−0.007 (−0.012, −0.002)	**0**.**010**
Arginine	0.02 (−0.008, 0.04)	0.19		
Aspartate	0.04 (−0.07, 0.15)	0.47		
Citrulline	0.04 (−0.008, 0.09)	0.10		
Glutamate	−0.02 (−0.05, 0.007)	0.14		
Glycine	0.0003 (−0.013, 0.014)	0.96		
Histidine	−0.03 (−0.06, 0.004)	0.09		
Leucine	0.002 (−0.02, 0.02)	0.88		
Ileleucine	−0.006 (−0.02, 0.006)	0.34		
Methionine	0.06 (−0.01, 0.1)	0.11		
Ornithine	−0.02 (−0.04, 0.007)	0.17		
Phenylalanine	0.02 (−0.03, 0.06)	0.47		
Proline	−0.01 (−0.02, −0.003)	**0**.**008**	−0.009 (−0.02, −0.0003)	**0**.**042**
Serine	−0.0008 (−0.03, 0.03)	0.96		
Tryptophan	0.03 (−0.02, 0.07)	0.28		
Tyrosine	0.02 (−0.02, 0.05)	0.28		
Valine	0.008 (−0.003, 0.019)	0.13		

CI, confidence interval; PA, pulmonary artery; GLS, global longitudinal strain.

*P* values less than 0.05 are highlighted in bold.

^a^ Multiple regression analysis on variables significant in univariate linear regression with adjustment for age, heart rate, and CVRF2 (hypertension, dyslipidemia, ever smoked).

We then conducted linear regression analysis between PA GLS and acylcarnitines (Table 4). Negative correlations were observed between C2, C4-OH, C12:1, C12, C12-OH/C10-DC, C14:3, C14:2, C14:1, C14, C14-OH/C12-DC, C16:3, C16:2, C16:1, C16:3-OH/C14:3-DC, C16:2-OH, C16:1-OH/C14:1-DC, C18:1-OH/C16:1-DC, C18-OH/C16-DC, C20:1-OH/C18:1-DC, C20-OH/C18-DC, and PA GLS. Multiple linear regression, adjusting for clinical covariates, was performed on PA GLS with significant acylcarnitines as predictor variables (Table 4). Medium and long-chain acylcarnitines remained significantly associated with PA GLS (C12, *P *= 0.027; C12-OH/C10-DC, *P *= 0.018; C14:2, *P *= 0.036; C14:1, *P *= 0.006; C14, *P *= 0.006; C14-OH/C12-DC, *P *= 0.027; C16:3, *P *= 0.019; C16:2, *P *= 0.006; C16:1, *P *= 0.001; C16:2-OH, *P *= 0.016; C16:1-OH/C14:1-DC, *P *= 0.028; C18:1-OH/C16:1-DC, *P *= 0.032).

**Table 4:** Summary of regression coefficients for PA GLS regressed on acylcarnitines.

Acylcarnitines	Unadjusted (Univariate linear regression)		Adjusted (Multiple linear regression)^a^	
	β (95% CI)	*P-*value	β (95% CI)	*P-*value
C2	−0.0003(−0.0006, −8*10^−6^)	0.044	−0.0003 (−0.0006, 7*10^−6^)	0.057
C4-OH	−0.07 (−0.13, −0.009)	0.025	−0.03 (−0.10, 0.03)	0.28
C12:1	−0.02 (−0.04, −0.001)	0.038	−0.02 (−0.04, 0.0001)	0.051
C12	−0.03 (−0.05, −0.007)	0.010	−0.02 (−0.04, −0.003)	**0**.**027**
C12-OH/C10-DC	−0.8 (−1.3, −0.3)	0.002	−0.6 (−1.05, −0.1)	**0**.**018**
C14:3	−0.3 (−0.5, −0.05)	0.017	−0.2 (−0.4, 0.03)	0.087
C14:2	−0.06 (−0.1, −0.01)	0.010	−0.05 (−0.09, −0.003)	**0**.**036**
C14:1	−0.04 (−0.06, −0.01)	0.002	−0.03 (−0.05, −0.009)	**0**.**006**
C14	−0.1 (−0.2, −0.05)	0.002	−0.1 (−0.2, −0.03)	**0**.**006**
C14-OH/C12-DC	−0.3 (−0.5, −0.1)	0.002	−0.2 (−0.4, −0.02)	**0**.**027**
C16:3	−0.4 (−0.7, −0.1)	0.004	−0.3 (−0.6, −0.05)	**0**.**019**
C16:2	−0.4 (−0.6, −0.2)	0.001	−0.3 (−0.5, −0.09)	**0**.**006**
C16:1	−0.1 (−0.2, −0.05)	0.001	−0.1 (−0.2, −0.05)	**0**.**001**
C16:3-OH/C14:3-DC	−0.8 (−1.5, −0.09)	0.026	−0.5 (−1.2, 0.1)	0.10
C16:2-OH	−0.5 (−0.9, −0.1)	0.007	−0.4 (−0.8, −0.08)	**0**.**016**
C16:1-OH/C14:1-DC	−0.5 (−0.8, −0.1)	0.005	−0.4 (−0.7, −0.04)	**0**.**028**
C18:1-OH/C16:1-DC	−0.4 (−0.7, −0.1)	0.004	−0.3 (−0.6, −0.03)	**0**.**032**
C18-OH/C16-DC	−0.2 (−0.4, −0.008)	0.041	−0.1 (−0.3, 0.08)	0.24
C20:1-OH/C18:1-DC	−0.2 (−0.4, −0.06)	0.008	−0.1 (−0.3, 0.03)	0.10
C20-OH/C18-DC	−0.2(−0.3, −0.02)	0.031	−0.1 (−0.2, 0.05)	0.18

PA, pulmonary artery; GLS, global longitudinal strain.

*P* values less than 0.05 in multiple linear regression are highlighted in bold.

^a^ Multiple regression analysis on variables significant in univariate linear regression with adjustment for age, heart rate, and CVRF2 (hypertension, dyslipidemia, ever smoked).

**Figure 2:**
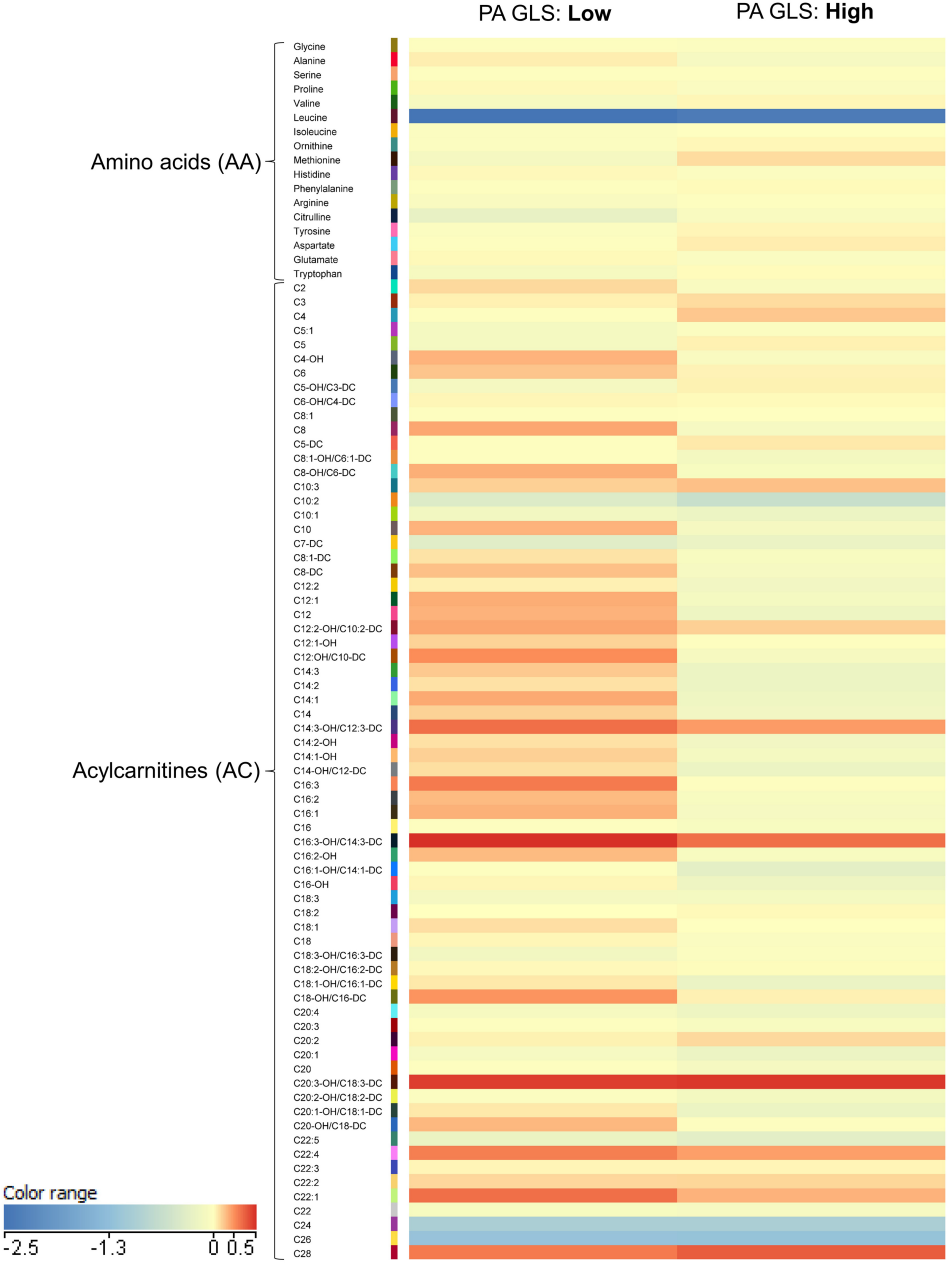
Heatmap illustrating metabolomics results in plot illustrating mean levels of key acylcarnitine.

A heatmap was generated to examine metabolite patterns in subjects with high vs. low PA GLS (Figure 2). Subjects with low PA GLS (indicative of high PA stiffness) exhibited higher levels of long-chain acylcarnitines, as evident from the increased prevalence of red for this class of metabolites on the heat map.

**Figure 3:**
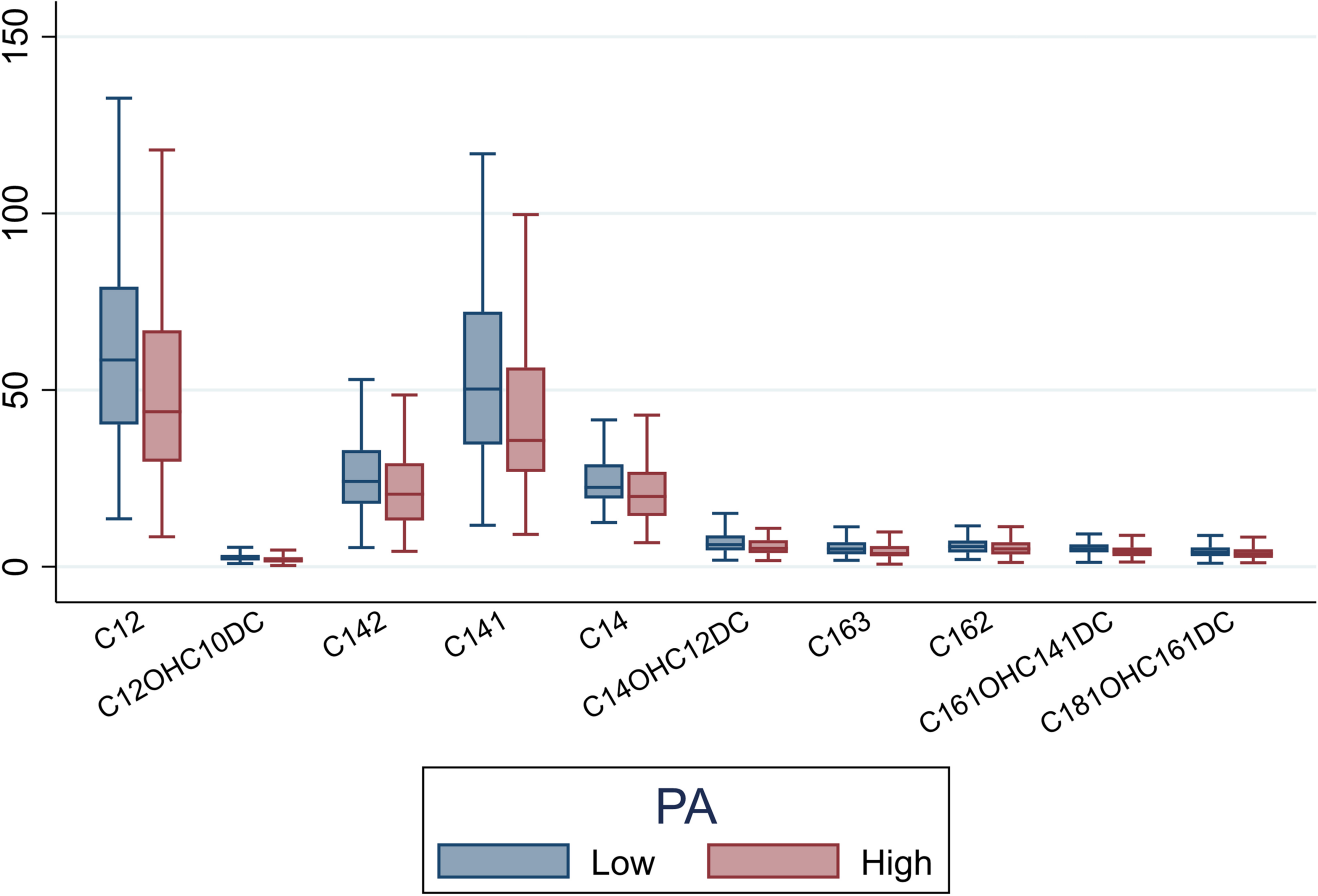
Box plot illustrating mean levels of key acylcarnitine metabolites that differentiate between high PA strain and low PA strain (*P *< 0.05 for all). PA, pulmonary artery.

We quantified levels of key metabolites that differed between participants with high vs. low PA strain (Figure 3). Participants with low PA GLS had higher levels of C12, C12-OH/C10-DC, C14, C14:1, C14:2, C14-OH/C12-DC, C16:2, C16:3, C16:1-OH/C14:1-DC, and C18:1-OH/C16:1-DC compared to participants with high PA GLS (*P *< 0.05 for all).

## 4 Discussion

PA GLS was evaluated as a novel method for assessing PA stiffness using conventional cine CMR images. Decreased PA GLS was characterized by specific clinical factors and metabolites among older adults. PA GLS was independently associated with aging, elevated heart rate, dyslipidemia, smoking, long-chain acylcarnitines, and amino acid metabolites.

### 4.1 PA GLS and clinical risk factors

This study lays the groundwork for future clinical applications of PA GLS in assessing PA stiffness. The correlation between PA GLS and clinical cardiovascular risk factors aligns with previous studies on PA stiffness (11, 26).

Age has been positively associated with PA PWV (11) and PA pressures (2, 5) in healthy subjects. Similarly, our study demonstrated that, like PWV and pressure, PA GLS decreased with age in a community cohort of older adults, indicating that aging is associated with longitudinal length changes in the PA.

Elevated heart rate is another risk factor associated with adverse cardiovascular events (27). In a porcine model, PA pressure was found to increase with heart rate, which was attributed to increase in flow and/or downstream flow resistance (28). A study of patients with pulmonary hypertension showed positive linear relationship between mean PA pressure and heart rate (29). Similar effects of heart rate on atrial distensibility were observed in earlier studies (30–32). PA distensibility was lower among patients with left heart disease and pulmonary hypertension vs. no pulmonary hypertension, and negatively correlated with heart rate (33). PA stiffness, assessed by the pulse pressure to stroke volume index ratio, has been shown to increase with heart rate (*r*^2 ^= 0.15), resulting in an increased risk of incident idiopathic PAH (26). Our study demonstrated an inverse association between PA GLS and heart rate (*r *= −0.24), which is consistent with the literature.

Smoking and dyslipidemia are crucial atherogenic risk factors. Smokers with chronic obstructive pulmonary disease often exhibit extensive atherosclerosis, leading to arterial stiffness (34, 35). Studies have shown that cigarette smoke induces increased wall stiffness in the PA of rats (36). Additionally, arterial stiffness has been directly associated with a 7-year increase in high-density lipoprotein, low-density lipoprotein, and triglycerides (37). Our study found that smoking and dyslipidemia were negatively associated with PA GLS, despite a small number of participants being at risk for dyslipidemia due to smoking.

### 4.2 PA GLS and metabolites

We integrated advanced CMR imaging with serum metabolomic signals to evaluate clinical features of PA stiffness and metabolite patterns among this aging cohort. GLS assessed stiffness representing the PA trunk, while serum metabolomics distinguished older adults with high vs. low PA stiffness. In our study, we observed an association between PA GLS and circulating amino acids and acylcarnitines.

Both alanine and proline were negatively associated with PA GLS in both univariate analysis and multivariable analysis adjusted for clinical CVRF2. Alanine, a non-essential amino acid, is strongly linked to glucose metabolism. It plays a prominent role in the glucose-alanine shuttle between muscle and liver. A small percentage of individuals older than 85 years and the overall population have reported echocardiographic signs suggestive of pulmonary hypertension, indicating elevated PA stiffness (38, 39). Studies have shown that alanine is positively correlated with the pulmonary arterial medial thickness index—a histologic marker of PAH (16), with higher levels in blood samples from the PA of systemic sclerosis patients with PAH (17). Increased alanine levels in these studies were attributed to reduced tissue alanine aminotransferase levels (17). Proline contributes to pulmonary arterial remodeling in PAH rats (40). Rafikova et al. found high circulating levels of alanine and proline in early-stage PAH patients (41).

Long-chain acylcarnitines increase with age in healthy individuals (42). Our previous study showed that medium- and long-chain acylcarnitines were independently associated with arterial stiffness (20), similar to left atrial strain patterns obtained via CMR (23). This aligns with other studies in patients with pulmonary vascular disorders that observed higher levels of circulating long-chain acylcarnitines in patients with PAH, including idiopathic PAH, chronic thromboembolic pulmonary hypertension, and pulmonary hypertension associated with left heart disease (43, 44). By demonstrating an association between long-chain acylcarnitines and PA strain in otherwise healthy adults, our observations suggest that such a metabolic underpinning may be present upstream in the absence of disease. These findings may support broader validation in other non-disease cohorts, providing strategies for screening or charting pulmonary vascular diseases.

Overall, our integrated data provide a fresh approach to phenotyping human cohorts with omics (45), allowing more in-depth characterization of pulmonary arterial function. In addition to omics data, other strengths of our study include the prospective nature of participant recruitment, reducing biases related to recall while facilitating the simultaneous acquisition of clinical, CMR, and metabolomics data. Our study was prespecified to characterize older adults without clinical cardiovascular disease; therefore, circulating biomarkers represent the aging process rather than disease processes.

### 4.3 Limitations

This study has limitations. Firstly, we did not acquire velocity-encoded flow measurements at the pulmonary trunk in this study, and are thus neither able to determine pulmonary vascular resistance noninvasively nor perform computational hemodynamic modeling for estimation of PA pressure. Secondly, compared to the current targeted approach, an untargeted metabolomics approach employing more metabolites may broaden the metabolic maps associated with PA GLS. While our targeted approach allowed precise quantification of identified metabolites, untargeted approaches could uncover pathways represented by these metabolites. Thirdly, all measurements and clinical variables originated from a single cross-sectional evaluation. Future studies may find it interesting to associate metabolomic findings at baseline with prospective changes in PA GLS and outcome data. Fourthly, while we did not explicitly exclude participants with preexisting respiratory disease, the calculated VO_2_ measurements of participants enrolled into our current study were generally within normal ranges, which suggests that the impact of clinically significant respiratory disease is likely to be low in this community cohort. Fifthly, the effect of physical activity on cardiorespiratory status impacting PA GLS measurements is uncertain in the studied subjects, although aerobic capacity was generally satisfactory among the subjects. Lastly, non-fasting serum samples (used in our study) may introduce analytic differences compared to other cohorts that use fasting samples. However, our practice aligns with other cohort studies that found fasting did not contribute to variability in most metabolite measurements (46).

## 5 Conclusions

Using conventional CMR, PA GLS was associated with aging and vascular risk factors among a contemporary cohort of older adults. Metabolic pathways involved in PA stiffness may include glucogenesis, collagen synthesis, and fatty acid oxidation. Among asymptomatic older adults, PA GLS may serve as a novel risk stratification tool to identify early metabolic perturbations associated with pulmonary dysfunction.

## Data Availability

The original contributions presented in the study are included in the article/Supplementary Material, further inquiries can be directed to the corresponding authors
