# Supplementary material for: Characteristics of pulmonary artery strain assessed by cardiovascular magnetic resonance imaging and associations with metabolomic pathways in human ageing
Source: Front Cardiovasc Med. 2024 Feb 29;11:1346443. doi: 10.3389/fcvm.2024.1346443 (PMC10937542; doi:10.3389/fcvm.2024.1346443)
Supplement: Supplementary file 1 [file Datasheet1.docx]

Supplementary Material

# Supplementary Figures and Tables

## Supplementary Figure


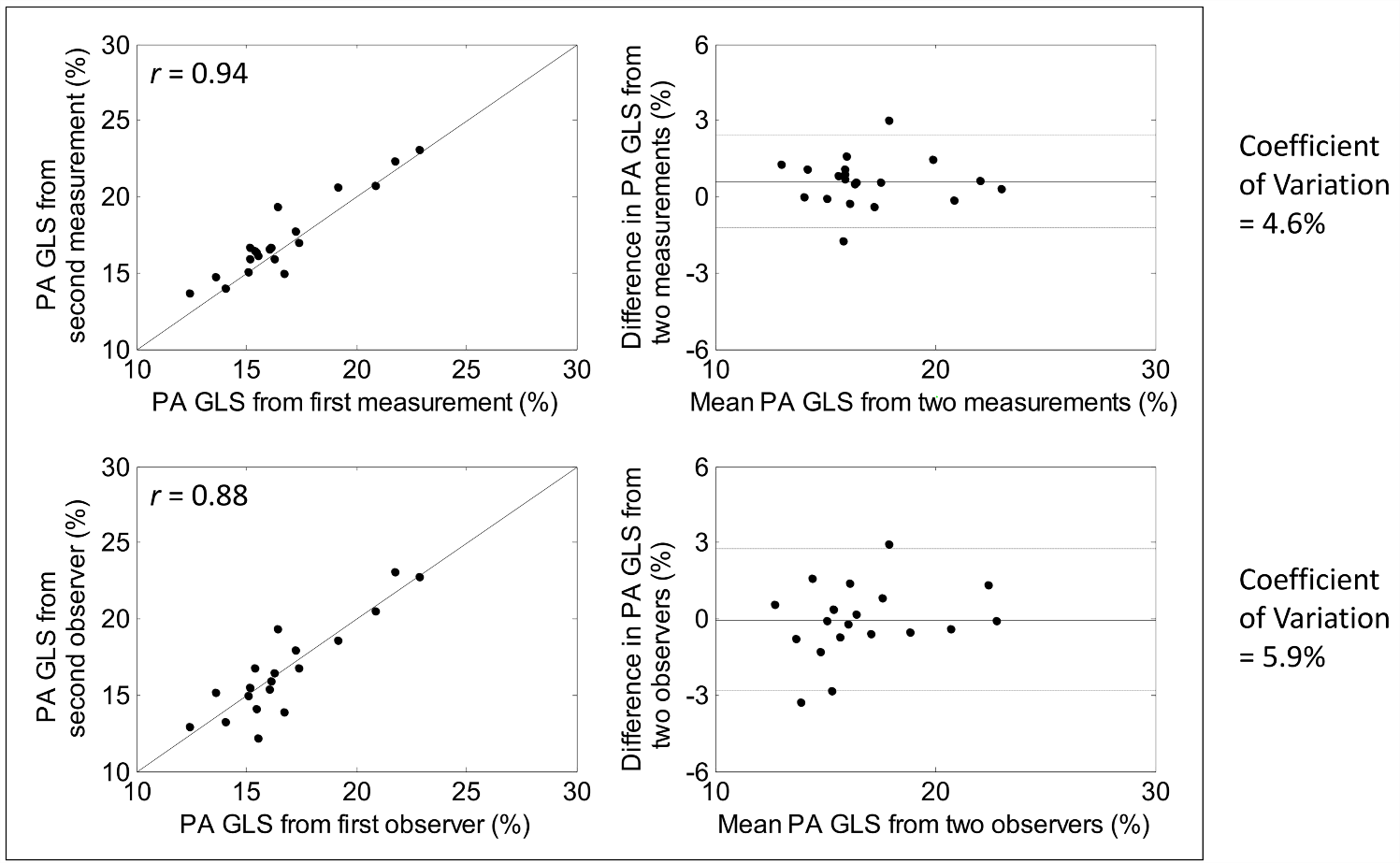


**Supplementary Figure S1.** Correlation and Bland-Altman plots showing (Top) intra and (Bottom) inter-observer agreement. PA GLS, pulmonary artery global longitudinal strain.

## Supplementary Table

**Supplementary Table S1.** List of measured metabolites

| **Short name** | **Name** |
| --- | --- |
| C2 | Acetyl carnitine |
| C3 | Propionyl carnitine |
| C4 | Butyryl carnitine or isobutryl carnitine |
| C5:1 | Tiglyl carnitine or 3-methyl crotonyl carnitine |
| C5 | Isovaleryl, 3-methylbutyryl carnitine, 2-Methylbutyryl, valeryl or pivaloyl carnitine |
| C4-OH | D-3-Hydroxy-butyryl carnitine, L-3-hydroxybutyryl carnitine |
| C6 | Hexanoyl carnitine |
| C5-OH/C3-DC | 3-Hydroxy-isovaleryl carnitine or malonyl carnitine |
| C4-DC/C6-OH | Methylmalonyl carnitine or succinyl carnitine |
| C8:1 | Octenoyl carnitine |
| C8 | Octanoyl carnitine |
| C5-DC | Glutaryl carnitine, ethylmalonyl carnitine |
| C8:1-OH/C6:1-DC | 3-Hydroxy- octenoyl carnitine or hexenedioyl carnitine |
| C8-OH/C6-DC | 3-hydroxy octanoyl carnitine or adipoyl carnitine, 3-methylglutaryl carnitine |
| C10:3 | Decatrienoyl carnitine |
| C10:1 | Decenoyl carnitine |
| C10 | Decanoyl carnitine |
| C7-DC | Pimeloyl carnitine, heptanedioyl carnitine |
| C8:1-DC | Octadecenedioyl carnitine |
| C8-DC | Suberoyl carnitine |
| C12:2 | - |
| C12:1 | Dodecenoyl carnitine |
| C12 | Lauroyl carnitine |
| C12:2-OH/C10:2-DC | - |
| C12:1-OH | Hydroxydodecenoyl carnitine |
| C12-OH/C10-DC | 3-Hydroxy-dodecanoyl carnitine or sebacoyl carnitine |
| C14:3 | - |
| C14:2 | Tetradecadienoyl carnitine |
| C14:1 | Tetradecenoyl carnitine |
| C14 | Myristoyl carnitine |
| C14:3-OH/C12:3-DC | - |
| C14:2-OH | 3-Hydroxytetradecenoylcarnitine |
| C14:1-OH | 3-Hydroxy-tetradecenoyl carnitine |
| C14-OH/C12-DC | 3-Hydroxy-tetradecanoyl carnitine or dodecanedioyl carnitine |
| C16:3 | - |
| C16:2 | Hexadecadienoyl carnitine |
| C16:1 | Palmitoleoyl carnitine |
| C16 | Palmitoyl carnitine |
| C16:3-OH/C14:3-DC | - |
| C16:2-OH | 3-Hydroxyhexadecadienoyl carnitine |
| C16:1-OH/C14:1-DC | 3-Hydroxy-palmitoleoyl carnitine or cis-5-tetradecenedioyl carnitine |
| C16-OH | 3-Hydroxy-hexadecanoyl carnitine |
| C18:3 | Linolenyl carnitine |
| C18:2 | Linoleyl carnitine |
| C18:1 | Oleyl carnitine |
| C18 | Stearoyl carnitine |
| C18:3-OH/C16:3-DC | 3-Hydroxyl-linolenyl carnitine or |
| C18:2-OH/C16:2-DC | 3-Hydroxy-linoleyl carnitine or hexadecadienedioyl carnitine |
| C18:1-OH/C16:1-DC | 3-Hydroxy-octadecenoyl carnitine or hexadecanedioyl carnitine |
| C18-OH/C16-DC | 3-Hydroxy-octadecanoyl carnitine or hexadecanedioyl carnitine, thapsoyl carnitine |
| C20:4 | Arachidonoyl carnitine |
| C20:3 | Dihomogammalinolenyl carnitine |
| C20:2 | - |
| C20:1 | - |
| C20 | Arachidoyl carnitine, eicosanoyl carnitine |
| C20:3-OH/C18:3-DC | - |
| C20:2-OH/C18:2-DC | - |
| C20:1-OH/C18:1-DC | Octadecenedioyl carnitine |
| C20-OH/C18-DC | 3-Hydroxy-eicosanoyl carnitine or octadecanedioyl carnitine |
| C22:5 | - |
| C22:4 | - |
| C22:3 | - |
| C22:2 | - |
| C22:1 | - |
| C22 | Docosanoyl carnitine, Behenoyl carnitine |

# Pulmonary Artery (PA) Global Longitudinal Strain (GLS) Measurement

PA longitudinal strain was measured using a semi-automated algorithm (1) that tracked the PA bifurcation and pulmonary valve in three-dimensional (3D) space:

Step 1: Landmarks on the PA bifurcation (obtained from the PA bifurcation view) and pulmonary valve annulus (six points, two each from the right ventricular outflow tract (RVOT), coronal RVOT, and RV 3-chamber views) were semi-automatically tracked over the cardiac cycle. The only user input was the landmark selection in the initial frame (2).

Step 2: Two-dimensional (2D) spatial coordinates of the aforementioned points were mapped onto a 3D coordinate system. This process utilized a transformation based on the Image Position, Image Orientation, and Pixel Spacing derived from the DICOM information:

$$\text{Coord}_{3D}=\mathrm{Coord}_{2D, x}\times\text{ImageOri}_{x}\times\mathrm{PixSpa}_{x}+\mathrm{Coord}_{2D, y}\times\text{ImageOri}_{y}\times\text{PixSpa}_{y}+\text{ImagePos}$$

where $\text{Coord}_{3D}$ represents the coordinate vector in 3D space, $\mathrm{Coord}_{2D, x}$ and $\mathrm{Coord}_{2D, y}$ denote 2D pixel coordinates in x- and y-axis, respectively; $\text{ImageOri}_{x}$ and $\mathrm{PixSpa}_{x}$ refer to the Image Orientation and Pixel Spacing along the x-axis, while $\text{ImageOri}_{y}$ and $\mathrm{PixSpa}_{y}$ are the Image Orientation and Pixel Spacing along the y-axis; $\text{ImagePos}$ denotes the Image Position.

Step 3: The above 2D-to-3D transformation was used to establish correspondence among different 2D cine images within the shared 3D coordinate system and to generate the 3D framework. The distance ($L$) between the PA bifurcation and the centroid of the pulmonary annular plane projected along the longitudinal direction was calculated within this 3D framework.

Step 4: The instantaneous PA longitudinal strain at a time point $t$ relative to an initial time point $t=0$ at end-diastole was calculated using the Lagrangian strain formula (Fig. 1B): $(L(t)-L(0))\times100/L(0)$.

Step 5: PA GLS was defined as the peak strain value (Fig. 1C).”

1. Zhong L, Leng S, Alabed S, Chai P, Teo L, Ruan W, et al. Pulmonary artery strain predicts prognosis in pulmonary arterial hypertension. *J Am Coll Cardiol Img*. (2023) 16:1022-34. doi: 10.1016/j.jcmg.2023.02.007
2. Leng S, Dong Y, Wu Y, Zhao X, Ruan W, Zhang G, et al. Impaired cardiovascular magnetic resonance-derived rapid semiautomated right atrial longitudinal strain is associated with decompensated hemodynamics in pulmonary arterial hypertension. *Circ Cardiovasc Imaging*. (2019) 12:e008582. doi: 10.1161/CIRCIMAGING.118.008582
